# Percutaneous screw fixation assisted by hollow pedicle finder for superior pubic ramus fractures

**DOI:** 10.1186/s12893-022-01659-z

**Published:** 2022-06-03

**Authors:** Hai Wang, Gui Wu, Chun-yong Chen, Yao-yu Qiu, Yun Xie

**Affiliations:** Department of Orthopaedics, First Affiliated Hospital of Fujian Medical University, Fuzhou, 350004 China

**Keywords:** Pubic ramus fracture, Cannulated screw, Fixation, Percutaneous

## Abstract

**Background:**

Pubic ramus fracture was an injury of anterior pelvic ring, the anterior pelvic ring plays an important role in maintaining the stability of the pelvis. The purpose of this study was to investigate the effect and indication of percutaneous retrograde pubic screw fixation assisted by hollow pedicle finder for pubic ramus fractures.

**Methods:**

The clinical data of 68 patients with pubic ramus fracture treated with cannulated screw from March 2008 to March 2020 were retrospectively analyzed. According to the surgical methods, they were divided into traditional surgery group (32 cases in group A, with traditional retrograde pubic screw fixation) and modified surgery group (36 cases in group B, with percutaneous retrograde pubic screw fixation assisted by hollow open circuit device). Operation time, blood loss, incision length, screw length and complications were recorded and compared between the two groups. On the second day after surgery, the maximum fracture displacement over plain radiographs, entrance radiographs and exit radiographs of the pelvis was evaluated according to Matta criteria to evaluate the postoperative fracture reduction. Majeed score was used to evaluate the hip function at 12 months after surgery.

**Results:**

The operations were successfully completed in both groups. The operation time, blood loss and incision length in group B were significantly less than those in group A (P < 0.05). There was no significant difference in screw length between the two groups (t = 0.797, P = 0.431). All patients were followed up for 8–38 months (mean 21.8 months). There were no vascular and nerve injury, fracture of internal fixator, screw entry into joint cavity, fracture nonunion and other complications in both groups. The fracture healing time of the two groups was 23.1 ± 2.1 weeks in group A while 22.7 ± 2.1 weeks in group B, respectively, and there was no statistical difference in the fracture healing time between the two groups (P > 0.05). In group A, there were 3 cases of incision infection, 1 case of incision fat liquefaction and 2 cases of lower extremity deep venous thrombosis, and the complication rate was 18.8%. There was only 1 case of lower extremity deep vein thrombosis in group B, and the complication rate was 2.8%, which was significantly lower than that in group A. The fracture in one case after surgery was found to be displaced in group A and no fracture was found in group B. There was no significant difference between the two groups in Matta imaging evaluation on the next day after surgery and Majeed function evaluation at 12 months after surgery (P > 0.05).

**Conclusion:**

Percutaneous retrograde pubic ramus screw fixation assisted by hollow pedicle finder is effective in the treatment of pelvic pubic ramus fracture. It has the advantages of less incision, shorter operation time, less blood loss and lower incidence of complications compared with traditional methods. However, correct surgical indications should be required when we apply this surgical method.

## Background

Pubic ramus fracture was an injury of anterior pelvic ring, the anterior pelvic ring plays an important role in maintaining the stability of the pelvis. At present, the surgical management of pubic ramus fracture injury can through both open approach and minimally invasive approach [1]. Although Open reduction and internal fixation (ORIF) could provide sufficient exposure of surgical field for bone fracture reduction and fixation, it has a lot of disadvantages, such as large amount of blood loss, huge soft tissue trauma and high incidence of complications. In addition, patients with pelvic fracture may accompany neurological, vascular, even urogenital and intestinal system injuries [2], so exploratory laparotomy and colostomy will usually be needed. If ORIF were performed after exploratory laparotomy or colostomy, the probability of incision infection will be further increased, and infection more often led to nonunion [3]. Minimally invasive surgery has obvious advantages for patients with pelvic injury. Percutaneous minimally invasive hollow screw fixation has been accepted by a lot of surgeons for patients with pubic ramus fracture for its biomechanical stability is comparable with that of plate fixation [4] and its low failure rate [5]. However, it was technically difficult to do closed reduction and lag screw fixation for a displaced pubic ramus fracture [1], as the conventional surgical instrument could not perform the fracture reduction and introduction of hollow screw guidewire simultaneously.

Hollow pedicle finder has been widely used in minimally invasive pedicle screw placement [6]. Both pedicle screw path preparation and guidewire introduction can be done via the hollow pedicle finder. To make percutaneous minimally invasive hollow screw fixation feasible for the treatment of displaced pubic ramus fracture, we use spinal hollow pedicle finder to assisted the reduction of pubic ramus fracture percutaneously, and then the introduction of hollow screw guidewire. In this study, we compared the effectiveness of this modified percutaneous surgical technique with the traditional open retrograde pubic screw technique in treating pubic ramus fractures.

## Patients and methods

### Patient selection criteria

The clinical data of patients with pubic ramus fractures treated by hollow screw fixation between March 2008 and March 2019 were analyzed retrospectively. According to the surgical methods, they were divided into traditional surgery group and modified surgery group. Inclusion criteria: ① Distal 1/2 fracture of the pubic ramus; ② The displacement of fracture is greater than 2 cm; ③ Closed fracture; ④ Percutaneous retrograde pubic screw implantation; ⑤ Fracture time < 3 weeks; ⑥ The follow-up data were completed. Exclusion criteria: ① Proximal 1/2 fracture of the pubic ramus which is not suitable for retrograde screw fixation [7]; ② Combined with local or systemic infection; ③ Combined with severe vascular or nerve injury; ④ Old ramus pubis fracture; ⑤ The obese. All patients in this study signed informed consent.

### Surgical procedure

Group A: Patients were in supine position after general anesthesia. An auxiliary incision was made at the fracture site to assist the reduction of fracture under direct vision, after the fracture reduction was completed, we use bone-holding forceps or scarf forceps to temporarily fix the fracture.

External and inferior of pubic tubercle was taken as the entrance point, the low-speed electric drill was used to slowly drill into the entrance point, X-ray fluoroscopy was performed to control the position of the implant. After the guidewire position was excellent, the hollow screw (diameter of 6.5 mm) with a suitable length was screwed in along the guidewire.

**Fig. 1:**
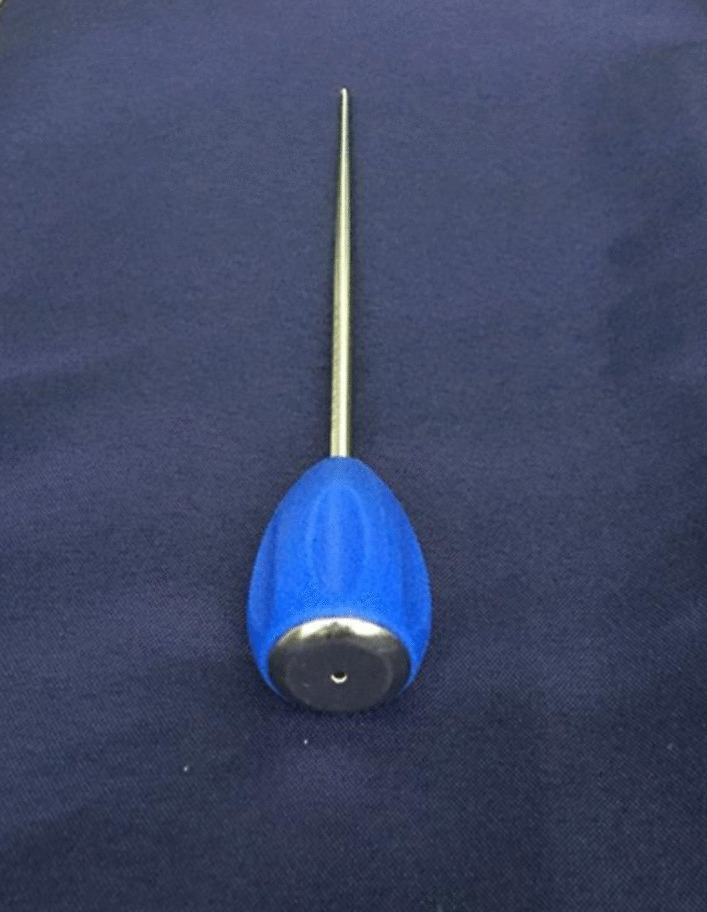
Appearance of the hollow pedicle finder. The external diameter of the anterior segment was 3.5 mm while the length of which was 15.0 cm; the external diameter of the posterior segment was 5.0 mm, and the length was 10.0 cm, and the total length was 25.0 cm. The inner core diameter was 2.0 mm

**Fig. 2:**
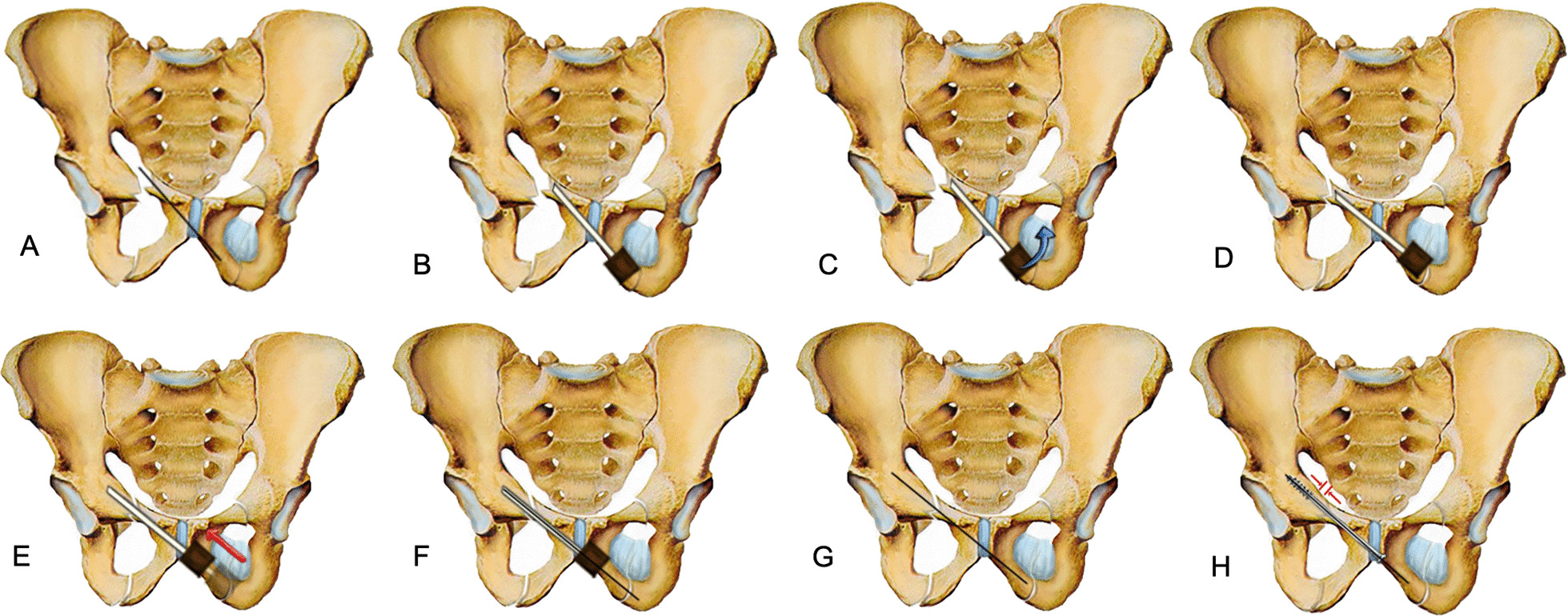
Schematic diagram of retrograde pubic screw implantation for closed reduction of pubic ramus fracture assisted by hollow pedicle finder. When the pubic ramus fracture was obviously displaced, if the fracture was not reduction before, the guiding needle of screw will be easily to penetrate from the fracture site, so most of the fractures will be needed to open reduction and temporary fixation before inserting the guiding needle of screw. This traditional surgical method has the disadvantage that we need to open reduction the fracture before guiding needle inserting; **B** The following is the schematic diagram of our modified surgical method. Place the hollow pedicle finder and adjust the direction according to the hand feeling, stop when the tip was close to the fracture site; **C** Percutaneous reduction the displaced fracture by leverage with the hollow pedicle finder, it was not necessary to pursue the anatomical reduction of the fracture at this time; **D** Initial reduction of the fracture was achieved by hollow pedicle finder; **E** Continue drilling into the hollow pedicle finder after fracture reduction; **F** When the hollow pedicle finder was in good position verified by C-arm X-ray, place the guiding needle into the hollow channel of the hollow pedicle finder and hammer the guiding needle gently to make it fix firmly; **G** The fracture will slightly displaced again when removing hollow pedicle finder and leaving the guiding needle alone, but we can reduction the fracture by inserting a cannulated screw according to the intramedullary reduction effect; **H** The fracture will be well reduction and pressurized when cannulated screw was screwed in

Group B: The parameters of the hollow pedicle finder used in the operation: the external diameter of the anterior segment was 3.5 mm while the length of which was 15.0 cm; the external diameter of the posterior segment was 5.0 mm, and the length was 10.0 cm, and the total length was 25.0 cm. The inner core diameter was 2.0 mm (Fig. 1). The patients were placed in supine position under general anesthesia. A 2–3 cm transverse incision was made from the joint of pubic symphysis to the superior branch of pubic symphysis to expose the entering point of lag screw. A 5 mm bone cortex was removed with a rongeur at the point of the entrance point. The direction was adjusted according to the hand feeling, if the resistance was fairly uniform and no sense of falling during entry, indicating that it did not penetrate the cortex in the cancellous bone channel. If the resistance was obvious or the resistance suddenly disappeared, it indicates that the angle and direction was wrong, and then the angle should be properly adjusted. If the fracture displacement was obvious (32 cases in this group), it is necessary to reduction the displaced fracture with the hollow pedicle finder to facilitate its smooth passage through the fracture end (at this time, there is no need to deliberately pursue the anatomical reduction of the fracture). When the position of the hollow pedicle finder is good over perspective, the inner core of the pedicle finder is needed to pull out, then the guidewire is penetrated into the hollow channel of the hollow pedicle finder and next the guidewire should be properly knocked to make it fix firmly. After the pedicle finder was pulled out, the hollow screw (diameter of 6.5 mm) with a suitable length was screwed along the guidewire. The hollow screw played an intramedullary effect to directly reduction and compress the fracture (Fig. 2).

### Postoperative management

At the second day after surgery, pelvic orthographic radiographs, pelvic inlet and outlet radiographs, and pelvic CT 3d reconstruction were performed to evaluate fracture reduction and whether the screw entering into the articular cavity. Patients without anticoagulant contraindications were routinely anticoagulant, and the patients were instructed to take ankle pump exercise function to avoid deep vein thrombosis. 6 weeks and 3, 6, 12 months after surgery, patients were asked to review the X-ray film, according to the review results to determine the weight-bearing walk or not.

### Observation indicators

The operation time, incision length, implant screw length, and complications of the two groups were recorded and compared. On the second day after surgery, the maximum fracture displacement on plain radiographs, radiographs at the entrance and exit of the pelvis was evaluated according to Matta criteria [8] to evaluate the postoperative fracture reduction, and Majeed score was used to evaluate the function at 12 months after surgery [9].

### Statistical analysis

Quantitative data were expressed as mean ± standard deviation (SD), and independent sample T test was used for comparison between groups. Counting data were expressed as rates, and *χ*^2^ test was used for comparison between groups. Wilcoxon rank sum test was used for comparison of grade data between groups. SPSS 22.0 was used for statistical analysis, and P < 0.05 was considered statistically significant.

## Results

Operation time, blood loss and incision length in group B were significantly less than those in group A (P < 0.05). There was no significant difference in screw length between the two groups (t = 0.797, P = 0.431).

All patients were followed up for 8–38 months (mean 21.8 months). There were no vascular and nerve injury, internal fixator fracture, screw entry into joint cavity, fracture nonunion or other complications in both groups. The bone union time of the two groups was 23.1 ± 2.1 weeks in group A and 22.7 ± 2.1 weeks in group B, and there was no statistical difference in the fracture healing time between the two groups (P > 0.05). In group A, 3 patients had wound infection and 1 patient had wound fat liquefaction, which healed well after anti-infective therapy and dressing change. In group B, no one had wound infection or wound fat liquefaction. 2 cases in group A and 1 case in group B developed deep venous thrombosis of lower limbs, which were cured after anticoagulation treatment. The complication rate in group A was 18.8%, higher than that in group B (2.8%). 1 case in group A was found to have re-displaced fracture at 6 weeks after surgery due to short screw implantation. Since the degree of displacement was not serious (< 2 cm), the patient was treated with conservative pelvic pocket external fixation and had regular follow-up review. After that, the fracture healed well and the function of the affected limb was not impaired. No fracture re-displacement occurred in group B.

**Fig. 3:**
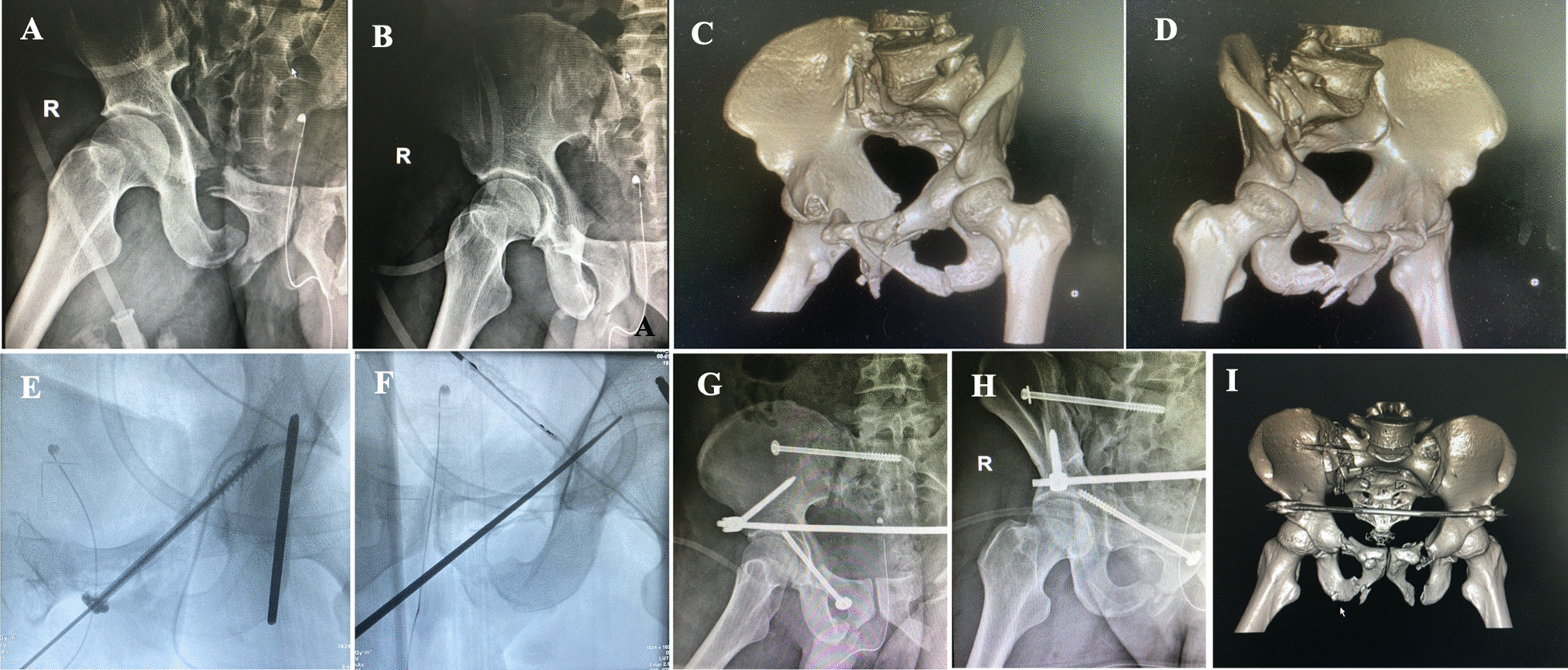
A 58-year-old male patient with pelvic fracture caused by falling from height, the displacement of right pubic ramus fracture was significantly greater than 2 cm, and surgical treatment was performed. Displacement of left pubic ramus fracture was less than 2 cm, and conservative treatment was performed. **A–D** Preoperative X-ray and CT film indicated displacement of the pubic ramus fracture is greater than 2 cm. **E** Intraoperative fluoroscopy indicated the pedicle finder entering a good bony channel and fracture obtained a initial reduction. **F** The fracture obtained a well reduction and compression when cannulated compressive screw was implanted. **G–I** Postoperative X-ray and CT film indicated the fracture was well reduction

There was no significant difference between the two groups in Matta imaging evaluation on next day after surgery or Majeed function evaluation 12 months after surgery (P > 0.05) (Tables 1, 2, 3). Typical case was shown in Fig. 3.

**Table 1:** General information of patients in the two groups

Group	Age (years)	Gender (cases)		Cause of injury (cases)			Tiles classification (cases)						Time from injury to surgery	Combined injury (cases)		
		Male	Female	traffic accident	high fall	others	A2	B1	B2	B3	C1	C2	(days)	Combined with craniocerebral, thoracic and abdominal injuries	Combined with colostomy	Combined with fractures in other parts
A	43.8 ± 4.8	19	13	13	16	3	2	9	6	6	5	4	9.5 ± 1.8	5	1	9
B	38.2 ± 5.2	20	16	18	13	5	5	9	6	6	6	4	9.8 ± 2.1	8	1	10

**Table 2:** Comparison of related clinical indexes between the two groups ($\overline{x }$ ± *s*)

Group	*n*	Operation time (minutes)	Blood loss (ml)	Screw length (mm)	Incision length (cm)	Fracture healing time (weeks)
A	32	46.3 ± 7.6	72.5 ± 10.8	67.8 ± 4.7	7.8 ± 1.1	23.1 ± 2.1
B	36	37.8 ± 6.6	31.0 ± 7.7	67.0 ± 4.9	3.4 ± 0.9	22.7 ± 2.1
Statistic		*t* = 4.890 *P* = 0.000	*t* = 18.038 *P* = 0.000	*t* = 0.797 *P* = 0.431	*t* = 16.772 *P* = 0.000	*t* = 0.823 *P* = 0.417

**Table 3:** Comparison of Matta imaging evaluation and Majeed function evaluation between the two groups

Group	Matta imaging evaluation				Majeed function evaluation			
	Excellent	Good	Fair	Poor	Excellent	Good	Fair	Poor
A	14	12	5	1	20	12	0	0
B	16	13	6	1	23	13	0	0
Statistic	*Z* = − 1.633 *P* = 0.102				*Z* = − 1.342 *P* = 0.180

## Discussion

Pubic ramus fracture was an injury to the anterior ring of the pelvis. It is generally considered that conservative treatment is feasible if pelvic stability is not affected or vascular, nerve and pelvic organ damage was not accompanied. With regard to surgical indications, it is generally believed that surgical treatment was recommended to reconstruct its stability when the displacement of the superior ramus pubis fracture is greater than 2 cm [10]. At this time, the stability of the anterior ring of the pelvis can be destroyed and fracture healing will be affected, so it requires surgical fixation. The traditional operation scheme was ORIF. However, due to its large trauma and large amount of blood loss, how to minimize the trauma to patients by minimally invasive surgery under the premise of ensuring the surgical effect is always the goal of orthopedic surgeons. With the development of computer navigation technology, minimally invasive pelvic anterior ring surgery can be achieved with its assistance, and the accuracy of intraoperative screw placement can be increased [11], however, these devices are usually expensive and the surgeons need a relatively long learning curve to handle the technique, making it difficult for primary hospitals to implement. At the same time, the main advantage of these navigation technologies is to improve the accuracy of nail placement, which cannot achieve the purpose of fracture reduction. A photodynamic bone stabilization system (PBSS) was reported to be a percutaneous operating method that provides intramedullary stabilization for pubic ramus fractures [12], however, it was just suitable for osteoporotic pelvic ring fractures. Therefore, how to improve the accuracy of screw placement, reduce the operation time and trauma to patients under the condition of closed reduction of pubic fracture is the key point and difficult point for orthopedic surgeons, and that is also the purpose of this study.

Compared with ORIF in the treatment of superior ramus pubis fracture, percutaneous internal fixation with hollow screw has significant advantages such as minimally invasive, less blood loss and early recovery [13]. However, the best indication for minimally invasive screw fixation is the fracture without displacement or only slight displacement [14]. For patients with obvious pubic fracture displacement, it is necessary to reduction the displaced fracture and maintain the reduction before screw guidewire placement, otherwise the screw guidewire will be easily to penetrate out the fracture site. Therefore, in most of the cases, it will be necessary to make an auxiliary incision for open reduction.

**Fig. 4:**
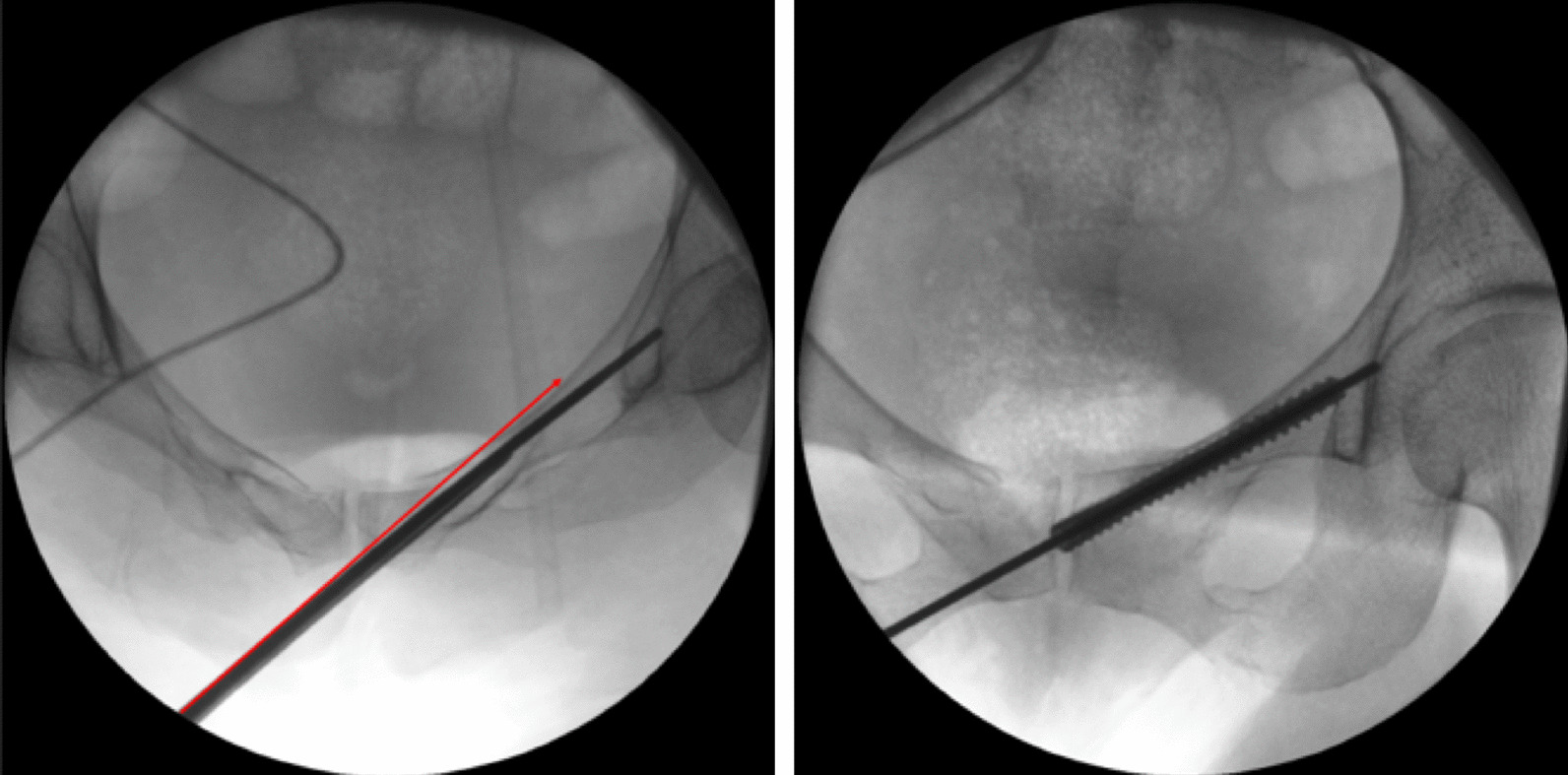
Schematic diagram of automatic deformation of the hollow pedicle finder. **A** In this case, the original channel of the pedicle finder was actually not the optimal channel, during the moving process of the pedicle finder, the tip of the pedicle was slightly deformed after touching the cortex, and it automatically entered the good channel instead of directly penetrating out of the cortex. The red line shows that if was the guide needle, due to its sharp tip, the guiding needle will directly penetrate out of the cortex and penetration into the pelvic cavity. **B** After the hollow pedicle finder automatically entering a good channel due to its slight deformation, we pull out the hollow pedicle finder’s inner core, and then kick into the screw guiding needle, and then pull out the pedicle finder. At last, we screwed the hollow screw directly follow the direction of the guiding needle, the screw entered a good channel

In addition, we also found that the traditional hollow screw fixation operation has the following disadvantages, which increase the difficulty and time of operation. Hollow screw guidewire is tiny and easy to deformation, once the guidewire was inserted into the unilateral cortex, it will be difficult to adjust the direction, and it will need multiple attempts of guidewire insertion thus the risk of injury to reproductive structures will increase [15]. The guidewire is sharp, and the tip will be easily to penetrate out the bone cortex during the operation. As the complex anatomical structure of the pelvis and acetabulum, irregular bone morphology, deep location and great variation, the surrounding important tissues and organs are dense, improper introduction of the guidewire would lead to vascular and nerve damage. Therefore, we modified the surgical method to perform surgery assisted by hollow pedicle finder. The high strength of the hollow pedicle finder can reduce the displacement of fracture percutaneously, and can also maintain the fracture reduction. Mosheiff et al. [16] found that the hollow screw inserted along the retrograding guidewire could further reduce fracture displacement via the intramedullary reduction effect, and we also found this phenomenon during the operation. The tip of the hollow pedicle finder is relatively round and blunt, and it is not easy to penetrate the cortex compared with the guidewire (Fig. 4). At the same time, because it is not easy to penetrate the cortex, it has a lower risk of iatrogenic injury to peripheral blood vessels, nerves and important organs. The results of this study showed that operative time, blood loss and incision length in group B were significantly reduced in group A, with statistically significant differences (P < 0.05). The hollow-pedicle-finder-assisted surgery can effectively reduce the difficulty and time of operation, and less trauma to patients, which provide a new technical choice for minimally invasive screw fixation treatment of pubic fracture.

We should pay attention to the problems as follows when we perform surgery with this technique: ① The bone window of the hollow pedicle finder at the entrance point needs to be slightly larger. If the window was too small, it will be too tight for the hollow pedicle finder to adjust the angle. Generally, we remove the cortex at the entrance point by the rongeur with a diameter of about 5 mm, slightly larger than the outer diameter of the pedicle finder. If the entrance point was too large it may cause too much damage to the cortex, which may affect the screw stability [17]. ② Before the pedicle finder entering, it is important to understand the fracture displacement mode in the horizontal and vertical directions, which can provide reference for the subsequent closed reduction assisted by the pedicle finder. ③ In the process of pedicle entering, if it moved in the correct channel, we can feel that the resistance was uniform. If the resistance is too large or the resistance suddenly disappeared, it indicated that the angle was not good. It was necessary to adjust the angle in time, or we need to confirm the angle of entry by fluoroscopy over the C-arm X-ray if necessary. ④ When the guidewire entered the inner core of the hollow pedicle finder and reached the bone, it was necessary to gently hammer the needle end to fix it securely, otherwise the guidewire may be taken out together with the hollow pedicle finder when we withdraw it out, thus it may increase the operation time. ⑤ Meng et al. [18] studied digital screw simulation through the 3D model of pelvis, and believed that the optimal entrance point for retrograde screw was the inferior horn of pubic symphysis. The average angles of screw entry which angled with sagittal plane, coronal plane and cross section were 51.10° ~ 53.44°, 8.24° ~ 8.45° and 39.47° ~ 35.26°. Mouhsine et al. [19] believed that the optimal entrance point for retrograde screw was slightly outside and below the pubic tubercle, with the tip aligned later and below the ipsilateral anterior superior iliac spine. The Angle between the insertion and the sagittal plane was about 45°, and the Angle between the transverse section was about 40°, and the insertion reached the upper margin of the acetabulum through the superior ramus of the pubis and the fracture site of the anterior column. Our experience was that the angle of needle insertion varies from person to person, and it can be adjusted according to the intraoperative situation after mastering the general angle. However, the selection of the entrance point was crucial. It was important to expose the bony marks around the entrance point with the electric knife. Otherwise, if the bone surface is not clearly exposed, the residual soft tissue will cause the misjudgment of the entrance point, which will lead to the failure to enter the optimal channel for screw implantation and even affect the subsequent fracture reduction. We usually choose the external and inferior of pubic tubercle as the entrance point. As for the selection of hollow screw diameter, it was demonstrated that percutaneous retrograde intramedullary screw fixation of superior ramus fractures with short smaller diameter screws is biomechanically inferior to fixation with longer and larger diameter screws, short 4.5-mm screws demonstrated increased dis- placement, lower stiffness, and decreased load to failure compared with all other screws [20], so it is generally recommended to use the diameter of 6.5 mm or 7.3 mm [21]. It was reported [18] that the average diameter of the narrowest area of the superior pubic ramus was (7.54 ± 1.02) mm in males and (6.23 ± 1.61) mm in females. In this study, all patients were fixed with 6.5 mm hollow screw, which was larger than the average narrowest area of the female pubic ramus, thus no splitting of the pubic bone was found.

Colostomy is a high-risk factor that can cause incision infection in pelvic fracture surgery, so we should pay attention to this. Before we start sterilizing, we usually use surgical drape to seal the area of colostomy and the edge curling up should be avoided. After paving the aseptic towel, we also suture the aseptic towel to the skin, only exposed the surgical area. Thus the colostomy area can be separated from the surgical area to avoid incision infection.

**Fig. 5:**
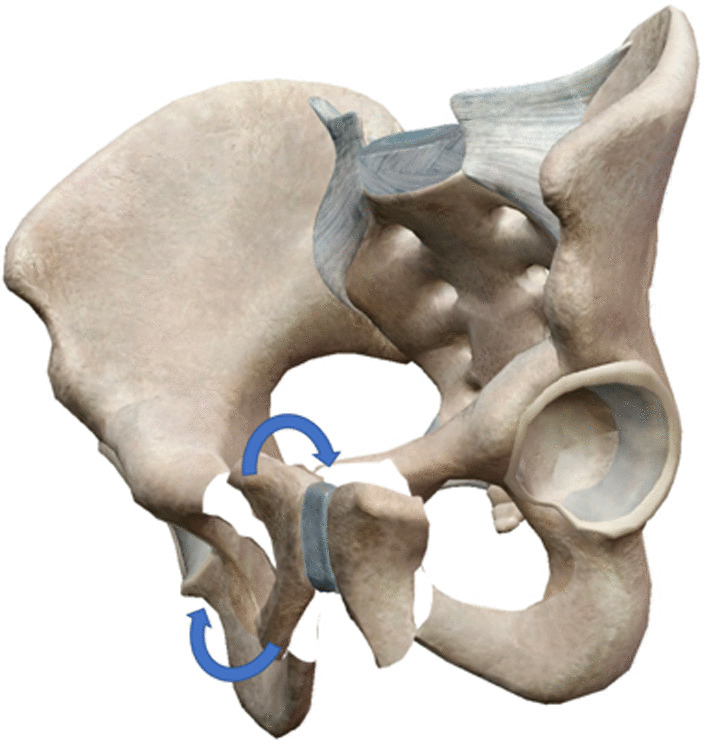
Sagittal pubic ramus fracture with rotational displacement. sagittal pubic ramus fracture with significant rotational displacement, which cannot be reduction by the hollow pedicle finder due to its cylindrical shape

This surgical technique was only suitable for distal 1/2 pubic ramus fractures, with or without fracture displacement. For the following fracture types, the author also conducted preliminary clinical exploration, however, we found that the effect was not ideal or could not be achieved. ① Sagittal fracture of the pubic ramus with obvious rotational displacement (Fig. 5). The author attempted to reduction 2 cases of pubic ramus fractures with significant rotational displacement at the sagittal level, but was unsuccessful, and at last we had to take ORIF to complete the operation. The possible reason for this we analysis was that because the hollow pedicle finder was cylindrical, it was suitable for reducing laterally displaced fractures and cannot reduction rotationally displaced fracture types. Therefore, this surgical technique is not recommended for reduction the fracture types with significant rotational displacement at the sagittal level. ② Old pubic ramus fracture: Because the callus of the old fracture has partially grown, it is extremely difficult to reduction it by hollow pedicle finder during the operation. The author has ever tried to apply the technique to 3 cases with old fracture, but unfortunately the reduction was not achieved, and finally we chose ORIF. Therefore, this surgical technique was not recommended in patients with old pubic ramus fractures. ③ Proximal 1/2 ramus pubis fracture. Retrograde screws were used for distal 1/2 fractures, while retrograde screws were used for proximal 1/2 pubic fractures. The entrance point of anterograde pubic screws located at the thickening area which between acetabulum to iliac wing, the distance between the bone entrance point to the skin incision was relatively far, and the soft tissue will block adjusting the angle, what’s more, the hollow pedicle finder from the proximal fractures, cannot be carried out on the displacement of fracture reduction, for displaced fractures is still need to open reduction, the advantages of closed reduction cannot be realized. The author tried to use this surgical method in 1 case of proximal pubic fracture with obvious displacement, however, due to the difficulty of reduction, the pedicle finder could not get into a good channel, and finally we had to apply ORIF. Therefore, this technique was not recommended for patients with proximal 1/2 pubic ramus fractures.

## Conclusions

Compared with traditional surgical methods, percutaneous retrograde pubic screw implantation assisted by hollow pedicle has the advantages of minimally invasive incision, less operation time and less blood loss. The main innovation of this study is that the closed reduction and screw placement of pubic displaced fractures can be realized with relatively simple tools, which avoids additional auxiliary reduction incision, simplifies surgical procedures and reduces surgical trauma to patients. Compared with traditional methods, this study has certain advantages such as avoid the high cost of equipment purchase, easy to promote and develop in primary hospitals. However, correct surgical indications should be required when we apply this surgical method.

## Data Availability

The datasets used and/or analyzed during the current study available from the corresponding author on reasonable request.
